# Challenges in conducting population-based seroepidemiology survey of COVID-19 in Lagos State, Nigeria

**DOI:** 10.1186/s12889-023-17125-1

**Published:** 2023-12-21

**Authors:** Adewale Kayode Ojogbede, Tajudeen Akanji Bamidele, Oluwagbemiga Aina, Toyosi Raheem, Azuka Okwuraiwe, Olufemi Amoo, Kazeem Adewale Osuolale, David Oladele, Abideen Salako, Fehintola Ige, Adesola Zaidat Musa, Ifeoma Idigbe, Fatimah Anwoju, Basit Baruwa, Hussein Abdur-Razzak, Bisola Adebayo, Kikelomo Wright, Aigbe Ohihoin, Oliver Ezechi, Rosemary Audu

**Affiliations:** 1 Public Health and Epidemiology Department, Nigerian Institute of Medical Research, Lagos, Nigeria; 2 Molecular Biology and Biotechnology Department, Nigerian Institute of Medical Research, Lagos, Nigeria; 3 Biochemistry and Nutrition Department, Nigerian Institute of Medical Research, Lagos, Nigeria; 4 Center for Human Virology and Genomics, Microbiology Department, Nigerian Institute of Medical Research, Lagos, Nigeria; 5 Monitoring and Evaluation Unit, Nigerian Institute of Medical Research, Lagos, Nigeria; 6 Clinical Science Department, Nigerian Institute of Medical Research, Lagos, Nigeria; 7 Lagos Bureau of Statistics (LBS), Ministry of Economic Planning and Budget (MEPB), Lagos, Lagos State Nigeria; 8 Department of Research and Statistics, Lagos State Ministry of Health, Lagos, Nigeria; 9 Department of Community Health & Primary Health Care, Lagos State University College of Medicine, Lagos, Nigeria

**Keywords:** Seroepidemiological study, Naso-oropharyngeal swabs, COVID-19, Lagos State

## Abstract

Population-based study is known to be a very essential type of study during and after a pandemic or epidemic, as it provides crucial information on the incidence, prevalence, and risk factors of the disease in question. There has been limited information about the challenges faced in conducting such surveys in Nigeria. In this paper, we will share our experience, and describe the challenges faced in conducting a population-based seroepidemiological study of COVID–19 in Lagos, Nigeria. Some challenges were peculiar to specific Local Government Areas (LGAs) while others were general. The challenges include general misconceptions of community members about health research, difficulties in mapping houses, planning for data collection, standardizing data collection, working in hard-to-reach communities when resources were limited as well as difficulty in collection of blood and naso-oropharyngeal swabs. Ways of overcoming these problems, lessons learnt, and recommendations are hereby discussed.

**Supplementary Information:**

The online version contains supplementary material available at 10.1186/s12889-023-17125-1.

## Background

The World Health Organization (WHO) declared the SARS-CoV-2 outbreak a Public Health Emergency of International Concern on 30 January 2020 [1] and was later declared a pandemic on 11 March 2020 [2]. Ever since then, this virus continued to spread rapidly and ravaged the entire globe leading to several cases and deaths. As of 18 April 2020, the spread of this emerging coronavirus infection, COVID-19, had caused over 2.1 million cases and over 146 thousand deaths worldwide [2].

In Nigeria, the first case of COVID-19 was confirmed on the 27^th^ of February 2020, and by the 19^th^ of April, 2020, 627 cases were confirmed, 21 confirmed fatalities, and 22 states of the federation were affected [3]. This disease continued to spread to all parts of the country despite the public health measures implemented by the government. Some of the measures included complete and partial lockdown, social distancing, the ban on large public gatherings including religious and social meetings, and dusk-to-dawn curfews.

Though COVID-19 testing was going on in the country, only individuals with travel history, contact with confirmed cases, and presence of symptoms were encouraged to test. However, a very high proportion of this infection had been reported to be asymptomatic [4, 5] which showed that community infection was driving the pandemic in the population. So, the actual burden of this infection in the community was not accurately reflected. A population-based sero-epidemiological survey was then recommended by the World Health Organization (WHO) to determine the burden of the disease in the community.

Subsequently, in 2020, a population-based seroepidemiological and household contact study of COVID-19 virus infection in Nigeria was conducted. However, the specific survey results and discussion are not presented here; instead, we describe the challenges encountered and how they were addressed during the pre-field and data collection periods of the sero-epidemiology survey conducted. Identifying challenges in data collection, logistics, sampling, and data analysis allows researchers and public health officials to refine their methodologies for future surveys. This process can lead to more accurate and efficient data collection techniques, ultimately improving the quality of epidemiological research. Also, understanding the challenges faced in resource-intensive surveys like seroepidemiology studies helps allocate resources effectively. Policymakers and funding agencies can prioritize investments in addressing specific bottlenecks to enhance the success of future research endeavors.

Furthermore, the challenges identified may have policy implications. For instance, if some issues were a significant challenge, policymakers might need to consider revising the approach to ensure better research outcomes. This can lead to more informed decision-making. Identifying challenges related to data collection and laboratory testing ensures researchers are aware of potential sources of bias or error in the dataset. This awareness can guide data quality control measures and improve the reliability of findings. Challenges related to community engagement could provide better information on community engagement strategies.

Finally, sharing experiences regarding the challenges faced in conducting a population-based seroepidemiology survey in Lagos State can contribute to a global body of knowledge. Other regions or countries facing similar challenges can benefit from these insights when planning similar studies. Therefore, the main aim of this study was to document all the challenges encountered during this sero-epidemiological and household survey in the epicentre, Lagos, Nigeria. This, we believe, could be valuable to other researchers conducting epidemiological research in similar terrains and settings.

## Methodology

### Aim

The aim of this study was to document all the challenges encountered during this sero-epidemiological and household survey in the epicentre, Lagos, Nigeria

### Study design and settings

This study was conducted in Lagos State, Nigeria which is a metropolitan city, the business and commercial hub of the country. The most populous state with over 20 million people, and administratively divided into 20 Local Government Areas [6]. It has the busiest airport (Murtala Mohammed International Airport) operating both local and international flights. Therefore, it serves as the main port of entry into the country. It was reported to have the highest COVID-19 cases in the country [7].

The sero-epidemiological and household contact study of COVID-19 was a population-based, age-stratified longitudinal study designed to collect data on the demographic, social, environmental, behavioural, biological, and serological factors that could contribute to the spread of the disease in the state. The WHO Generic protocol for a population-based, age- and gender-stratified sero-survey study for SARS-CoV-2 was used [8]. Participants were enrolled from all the 20 Local Government Areas (LGAs).

### Study population

The study population consisted of all selected household members, irrespective of age. Socio-demographic data and biological specimens such as blood and naso-oropharyngeal samples were collected from study participants.

#### Inclusion criteria

All persons living in the household were invited to participate in the study, including children to ensure the calculation of age-specific attack rates.

#### Exclusion criteria

Household members who were in residential institutions, such as boarding schools, dormitories, hostels, or prisons were excluded. Refusal to give informed consent, or contraindication to venipuncture also constituted an exclusion criterion.

### Sample size determination and sampling procedure

The sample size of 2400 participants for the study was determined by the Cochran formula using the prevalence of 5.5% calculated based on the positivity rate of COVID-19 cases tested at the Nigerian Institute of Medical Research (NIMR), α = 0.05, where ${Z}_{\frac{\propto }{2}}$  = 1.96 and the margin error (e) is specified at 1%. The needed number (n) of participants was therefore estimated using a 2-tailed test at level α given by:

$$n=\frac{{Z}_{\frac{{\propto }^{2}}{2} p {\left(1-p\right)}^{2}}}{{e}^{2}}$$

where ${Z}_{\frac{\propto }{2}}$ is the standardized normal deviate corresponding to the levels of the defined values of α. 1.0 design effect was used, and a 20% non-response rate was also added.

A total sample size was estimated as:

$$\begin{array}{c}n=\frac{{{1.96}^2X0.055\left(1-0.055\right)}^2}{{0.01}^2}\\\mathrm n=1886.85\end{array}$$

20% non-response rate = 377.

The total sample size required with 1.0 design effect is approximately 2400 participants for the study.

A proportionate sampling technique was used to select participants from all the LGAs in the study. Thirty Enumeration Areas (EA) were selected from the 20 LGAs based on the prevalence of COVID–19 in the LGAs at the time. Twenty households were selected randomly from each EA making a total of 600 households (HHs) for the state and 2400 total samples provided the average HH size of four for the state according to the National Population Commission (NPC).

### Data collection

Information on the socio-demographics, exposure information, clinical information, and drug history was collected from all participants recruited into the study. Study-related information was collected by 60 trained research assistants using electronic data collection tools (RedCap). Where the electronic tool was not available, hard copies of the data collection tool were used. Data on exposure history and clinical symptoms were collected on follow-up visits—days 7, 14, 21, and 28.

### Specimen collection

Fifteen millilitres of venous blood were collected from each participant into plain vacutainer bottles. The blood samples were allowed to stand at room temperature briefly before the sample was centrifuged and the serum collected into clean cryovials. The serum samples were stored at -20 °C until use. The blood sample collection was done thrice in the study; at enrolment and at days 21 and 28.

Naso-oropharyngeal swab samples were collected from all contacts at baseline and at the four follow-up visits. Once collected, swab samples were immediately placed into viral transport mediums (VTM) and stored in a refrigerator (2—4 °C) at the state level before transportation to the designated reference laboratory. Samples were transported from state capitals to the referral laboratory using the NCDC Tranex courier system. Appropriate IPC and biosafety measures followed for sample collection and transportation.

### Specimen transport

All those involved in the collection and transportation of specimens were trained on safe handling practices and spill decontamination procedures. Sample transport and infection control advice with respect to this study was in accordance with the case management algorithm of the Federal Ministry of Health [FMoH] [9].

For each biological sample collected, the time of collection, the conditions for transportation, and the time of arrival at the study laboratory were recorded. All samples collected were shipped to the Center for Human Virology and Genomics at the Nigerian Institute of Medical Research, Yaba, Lagos. This laboratory is ISO 15189 accredited and is also a WHO-listed prequalification laboratory. The specimens were transported to the designated laboratory as soon as possible after collection. In occasions where the specimens were not likely to reach the laboratory within 72 h, specimens were frozen at -20 °C and shipped on dry ice by an IATA-certified courier company.

### Laboratory evaluations

Laboratory and biosafety guidance for COVID-19 was done according to the WHO guidelines [10]. Serologic assays listed by WHO were used for this evaluation. The serological test kits utilized were Euroimmun anti-SARS-CoV-2 nucleocapsid protein [NCP] immunoglobulin G [IgG], which was an ELISA method and Abbott Architect SARS-CoV-2 IgG, a chemiluminescent microparticle immunoassay technique. Both assays were testing for IgG. The tests were carried out according to the manufacturer’s direction [11]. The laboratory procedures involving sample manipulation were carried out in a biosafety cabinet in a level 2 laboratory.

### Serological testing

Serum samples were screened for the presence of SARS-COV2 antibodies. Two different serological tests for IgG were carried out using an enzyme-linked immunosorbent assay (ELISA) that has been appropriately validated [11].

### Nucleic acid amplification and testing

Swab samples were inactivated according to national protocol and the nucleic acid was extracted using a Qiagen kit. Extracted nucleic acid was subjected to qPCR testing using a nationally recommended kit. The results were interpreted in accordance with the national algorithm. Positive cases were referred to the nearest treatment center for further management and the first few X cases and contacts (FFX) investigation protocol for coronavirus disease 2019 (COVID-19) (FFX protocol) was activated.

### Sample storage

In the case that serum samples could not be processed immediately, they were stored at -80 °C. Samples were aliquoted prior to freezing, to minimize freeze–thaw cycles. The remaining samples were stored in NIMR biorepository at -80 °C.

### Community entry and data process

Before the fieldwork, an advocacy visit was paid to all the key stakeholders in each selected community – gatekeepers (i.e*.* community heads locally called* ‘*Baale’), Community Development Association (CDA) chairmen, etc., and meetings were held with Medical officers of Health and Health Educators in each LGA. Mappers enumerated all the selected communities to have the details of the current occupants. Community sensitization preceded community entry in all the LGAs. Fifteen teams of field workers were then dispatched to the 30 EAs for data and sample collection, hence a team was responsible for two EAs. A team comprised one data collector, two sample collectors, one mapper, and one community guide. The mapper doubled as the house tracker and data collector. There were supervisors who monitored members of the research team in each LGA. Supervisors also documented and reported incident(s) on the field.

A three-days training session was held for all field workers; data, and sample collectors. They were trained on the objective of the survey. The data collectors were trained on how to collect quantitative data using the study questionnaire and the meaning of each of the questions was taught, while the sample collectors who were phlebotomists were trained on effective means of sample collection and management in the field according to the study protocol. Subsequently, a one-day dry run was also conducted on the third day of the training in Gbobi Sabe area of Lagos Mainland LGA to examine the feasibility of the main survey, and competence of field workers and to pre-test the data collection tools for validity and reliability. The field workers were grouped into teams. Each team comprised two data collectors and two sample collectors. Data and samples were collected from all individuals who consented in the area on that day. Errors were noted in the questionnaire and were later corrected. Then the main survey commenced.

In each household where consent was obtained, a baseline data was collected from all members of the household. Also collected were blood and naso-oropharyngeal swab samples. A follow up visit was conducted in any household with both positive and negative COVID-19 cases in order to determine the transmission rate.

The follow-up visits were conducted on days 7, 14, 21, and 28. For the day 7 and day 14 follow-up, only naso-oropharyngeal swabs were taken from all the negative members while blood samples and naso-oropharyngeal swab samples were collected on days 21 and 28. Incentives such as milk, hand sanitizers, and detergents were given to participants to encourage participation. Dispatch riders picked samples from the field (primary health centers/LGA headquarters) every evening to the laboratory at NIMR.

## Challenges and solutions

### Challenges during mapping and enumeration

#### Lack of identification of the mapper

During the mapping and enumeration exercise, the mappers had no means of identification such as ID cards, reflective jackets, or overall gowns indicating the name of the survey. This affected participation negatively as many identified participants were not convinced due to the lack of identification of the house mappers.

##### Solution

Most of the mappers recruited for the exercise were staff of the National Population Commission (NPC). So, they presented their NPC staff identity cards and persuaded the participants on the importance of the enumeration to the spread of COVID-19 in the state.

#### Refusal to participate and poor mobilization and sensitization

Many households refused to provide their details and be enumerated because of poor sensitization, and mobilization in the community. Many complained they were not aware, and others did not see anything good in government programmes and were angry with the government because of how degenerated the economy had become, making living difficult for the people.

##### Solution

The Health Educators for the LGAs and the chairman of CDAs were notified about the refusals despite prior sensitisation. They mobilized the association of all landlords and landladies in those communities who then worked together to further sensitise and create better awareness to motivate participants in the communities. In addition, the Health Educators provided community guides in each community, who were members of the community, to inform people about the exercise and to accompany the mappers as they moved from house to house. Furthermore, the CDA chairmen held meetings with members of the community to inform and encourage them to participate in the survey.

Research team members and community mobilizers also took time to educate members of the community on what they stand to benefit from the outcome of the research in terms of quality of healthcare and not direct financial gain to individuals or groups of individuals. This helped to address the general misconceptions of community members about the research.

#### Houses under lock and key

Many houses were locked up during the visit. The occupants were already out for work. Lagos being a congested metropolis with heavy traffic most of the time, many residents leave for work very early and return late at night; hence, they are not available during the daytime.

##### Solution

The occupants of these houses were revisited in the evenings and some others during the weekends.

### Challenges in both urban and rural communities

#### High cost of lodging for field workers

Places such as Badagry, Epe, Ibeju -Lekki, Lekki, and Ikorodu were far from the city center and NIMR, so, it was impossible for workers to go to the field and return home the same day. Therefore, it was expedient to lodge the field workers somewhere safe and close to the field, however, the study was faced with limited resources making it difficult to lodge them in comfortable hotels.

##### Solution

The field workers were lodged in pairs of the same-sex in safe hotels close to the field.

#### Restocking incentives in hard-to-reach areas

Incentives were given to the participants as motivation but restocking it when it got finished in the hard-to-reach areas was a challenge. There was difficulty in going back on the water, on bad roads, and on the bike to carry those incentives risking life and wasting significant time. This made many participants lose interest in participating in the survey.

##### Solution

The study challenge was fully explained to the participants.

#### Fear of being injected with COVID-19

The study was conducted immediately after the ease of the lockdown when COVID-19 spread, and deaths were still on the increase. Also, during this period many online videos were circulating about injecting people with COVID-19. So, it became a great challenge for the field workers as many believed they were injecting residents with COVID-19, causing a lot of refusals.

##### Solution

To mitigate this challenge, the field workers presented their ID cards, letters of introduction, and approval from NIMR and relevant government authorities explaining what the study was all about.

#### Refusal for sample collection

Refusal for collection of blood and naso-oropharyngeal samples was also a big challenge. Some people were interested in the study but were discouraged when it came to sample collection. These participants were ready to answer questions in the questionnaires but refused sample collection; some said it was painful, and some were afraid their samples would be used for rituals.

##### Solution

Incentives were the only bailout for this challenge. Some participants had a re-think because of the incentives – hand sanitizer, sachet milk, and detergents since it was a condition to get the incentives.

### Challenges in the rural

#### Accessing hard–to-reach communities

Collecting data in some areas of Lagos State was difficult and frustrating. Some communities in the rural areas were hard-to-reach. These places are extremely far from the city with very bad roads, some are not reachable by vehicle except by motorcycle while some can only be reached by canoe. These include Iji, Agodo, and Origorigan communities in Epe LGA which were far, with no motorable road access; while Ilado-Tomaro in Amuwo-odofin LGA could not be reached by vehicle, tricycle, or motorcycle only through a canoe ride.

##### Solution

For Iji, Agodo, and Origorigan, the motorcycle was the only option to access the community. The field workers had to transport themselves to the place on motorcycles, carrying all data collection materials and incentives over several hours to the places while for Ilado-Tomaro canoe was used to convene field workers and materials to the area. In addition to this, some of the field workers were aquaphobic.

#### Hostility by the residents

Some communities in Agege, Ifako Ijaye, Ibeju Lekki, and Epe LGAs were very hostile towards the field workers. They were almost attacking the field workers because these residents believed field workers were from the government, which had neglected them due to lacks of basic amenities such as good water supply, constant power supply (electricity), good roads, etc.

##### Solution

The community guides were highly instrumental in calming the residents down as they were indigenes of the same community. They explained to them the importance of the survey and that the field workers were not directly from the government and had nothing to do with their agitation.

## Discussion

The challenges faced during planning and conducting population surveys could vary depending on several factors or circumstances at hand such as level of funding, terrain of the area, security level of the study site, cooperation of the stakeholders, community perception of research, previous experience of community members on research/field activities, cultural belief of the community members, approach and experience of the research teams, and many more. Our experience in implementing a population-based epidemiological survey involved several challenges such as poor mobilization and sensitization, selected houses under lock and key, fear of being injected with COVID-19, poor participation, blood draw refusals, difficulties in accessing hard-to-reach communities, hostilities from community members, high cost of lodging in some areas.

Poor participation experienced among the community members in this study could be traced to several factors. Firstly, sensitization and mobilisation, play a key role in any community or population survey. The more people are sensitized and mobilised, the greater their understanding of the research objectives, and their participation. Sensitisation must be done properly ahead of the fieldwork or survey data collection – informing and dialoguing with all necessary stakeholders, e.g., kings, baale, LGA chairmen, medical officers of health, CDA chairmen, etc., who would, in turn, encourage their community members to participate. Unfortunately, this was not extensively done in this study due to poor funding. In line with this, a study conducted in Uganda observed an overall 20% increase in knowledge, a 50% increase in awareness levels among those with little or no education, and by 41% among young people (15–24 years) about adverse drug events in the community after implementation of the community dialogue and sensitization (CDS) program compared to before the program began [12]. Furthermore, a combination of approaches to mobilize and seek consent from the community evidently increased participation and success in the study, and this agreed with the reports of other previous researchers [13–18].

Secondly, the study was conducted immediately after the ease of COVID-19 lockdown. So, there was fear that our data collection team might inject them with the COVID-19 virus following the myths flying around at the time. This gave us a great setback in data collection as many participants doubted our genuineness and subsequently refused participation.

Thirdly, poor timing contributed to poor participation. Most oftentimes during the day in Lagos, houses are under lock and key, and residents are not at home; either at work or school or market, etc. These necessitated revisiting these houses either late in the evening or during the weekends, which was not always possible for all the houses. This observation agreed with the report of a study that stated that the timing of research involving households plays a crucial role in achieving a high response rate in community-based research [19]. The revisiting impacted adversely on the study resources (man hour, finance), and the team had to expend efforts in working for longer hours on weekend in the affected areas.

Fourthly, the slight discomfort or irritation experienced by participants during sample collection (especially naso-oropharyngeal swabs) greatly discouraged participation, especially when there was a lack of perceived immediate benefits for them. Extra efforts were made to convince them by providing incentives for participants. In support of this, the nature of the study (type of biological sample collected) and perceived benefit would influence participation [19].

Furthermore, the coverage or response rate in the city was lower compared to the rural communities. This is because there are less interactions in the neighbourhood in the cities compared to the rural ones. People doubt the intentions of anyone who knocks on their door, as there’s a perception of reduced security in the city. Many believed they were educated and had personal/family physicians, thus needing no medical help. This led to outright refusal without listening to the research teams or reading of participant’s information/consent form.

Hostility encountered in some communities at Agege, Ifako Ijaye, Ibeju Lekki, and Epe LGAs was understood to be caused by a lack of trust in the government, perceived neglect from the government, and fear of strangers coming to the community. It was a time when most people were not allowed to work or had just lost their jobs due to the pandemic, no work, no salary (except the government workers – civil servants). So, it was a really tough and hard time for many Nigerians.

Expectations of community members from the government were high in terms of COVID-19 palliatives such as food, financial donations or allowances, etc., which most did not get, making people infuriated with the government. Seeing research staff move from house to house exacerbated their anger. They believed that the government was doing a wrong thing at that time.

Additionally, researchers’ fatigue is inevitable in this situation; interviewing people and collecting samples from house to house, street to street is no doubt fatiguing [20]. An aggravating factor is the low response rate among community members, walking a long distance under the sun, no vehicle attached to individual teams, and many more. Fortunately, there were no security challenges compared to some studies conducted almost at the same time in the northern part of Nigeria, where insurgencies—Boko Haram, kidnapping etc. were reported as threats and great challenges [20, 21]. Although Security agencies (e.g. police, neighbourhood security) were duly informed ahead of the fieldwork (for the provision of adequate security), there were also local guides (community members), allocated to accompany each team.

Also, contrary to the study in northeast Nigeria, gadget issues such as battery running low or no power on the data collection devices were not challenges, as all teams were provided with a power bank as a backup in our study [20]. Furthermore, other challenges such as culture and religion (i.e. lack of cultural/religious sensitivity), poorly informed consent procedures, gender issues, language barriers, and previous bad experiences [18] were not challenges in this study, as each team was carefully selected considering religion, gender, spoken language, etc., and was also thoroughly taught informed consent procedures.

Finally, research funding is paramount, and impacts nearly all areas of research, such as planning, training, logistics, transportation, remuneration, laboratory analysis, etc. the paucity of funding is a challenge in this research that cannot be overemphasized.

Many resources for the study were inadequate; hence, low remuneration for field workers, the research teams could not be accommodated in standardized hotels near the study sites, nylon raincoats were improvised as raincoats, no rain boots were provided, remuneration for research staff affected, mobilization and sensitization were not rigorously and properly done, mappers’s ID cards were not provided. A similar report linking inadequate funding of field research as an important challenge had been reported previously [19].

## Conclusions

The sero-epidemiology survey conducted in Lagos State was an important exercise and it was very successful. However, there were many challenges that were faced during the exercise. These ranged from low funding, difficulty during mapping and enumeration, refusal to enrol and participate by some community members, poor mobilization and sensitization, locked houses at the time of visitation, high cost of lodging for field workers, difficulty in restocking incentives in hard to reach areas, fear of being injected with COVID-19 and the consequent refusal of sample collection as well as hostility by the residents towards research team members. However, the commitments and experience of the research team members assisted in proffering solutions to most of the challenges, and hence a successful community survey was conducted.

### Supplementary Information

**Additional file 1.**

## Data Availability

The data generated from this study is stored in REDCap database, and available upon request from Rosemary Audu, Principal Investigator (rosemaryaudu@yahoo.com).

## Abbreviations

CDA: Community Development Association
CDS: Community Dialogue and Sensitization
EA: Enumeration Areas
HHs: Households
LGAs: Local Government Areas
NCDC: Nigeria Centre for Disease Control
NIMR: Nigerian Institute of Medical Research
NPC: National Population Commission
WHO: World Health Organization

## References

[1] WHO. COVID-19 Public Health Emergency of International Concern (PHEIC) Global research and innovation forum. 2020a. www.who.int/publications/m/item/covid-19-public-health-emergency-of-international-concern-(pheic)-global-research-and-innovation-forum. Accessed 5^th^ Sept 2020.

[2] WHO. Coronavirus disease 2019 (COVID-19) Situation Report – 89. WHO. 2020b. https://www.who.int/emergencies/diseases/novel-coronavirus-2019/situation-reports. Accessed 25^th^ July 2020

[3] NCDC. COVID-19 Outbreak in Nigeria Situation Report - 15. 2020. Accessed 5^th^ Sept 2020.

[4] Day M (2020). Covid-19: four fifths of cases are asymptomatic China figures indicate. BMJ.

[5] Kumar N, Hameed SK, Babu GR, Venkataswamy MM, Dinesh P, Bg PK, John DA, Desai A, Ravi V (2021). Descriptive epidemiology of SARS-CoV-2 infection in Karnataka State, South India: transmission dynamics of symptomatic vs. asymptomatic infections. EClin Med.

[6] Idris J, Fagbenro A. Lagos the Mega-City: A report on how the metropolis handled an outbreak of the Ebola epidemic. Socio Cultural Dimensions of Emerging Infectious Diseases in Africa. 2019:281–98. 10.1007/978-3-030-17474-3_21. PMCID: PMC7123743.

[7] NCDC. COVID-19 updates state report. 2020b. https://covid19.ncdc.gov.ng/report/. Accessed 5^th^ Sept 2020.

[8] World Health Organisation. The unity studies: WHO sero-epidemiological investigations protocols. n.d. Retrieved from https://www.who.int/emergencies/diseases/novel-coronavirus-2019/technical-guidance/early-investigations.

[9] Nigeria Centre for Disease Control guidance for laboratory testing of novel coronavirus (COVID-19). 2020.

[10] World Health Organization. Guidance for laboratories shipping specimens to WHO reference laboratories that provide confirmatory testing for COVID-19 virus: interim guidance. 2020. p. 31.

[11] Ige F, Hamada Y, Steinhardt L, Iriemenam NC, Uwandu M, Greby SM, Aniedobe M, Salako BL, Rangaka MX, Abubakar I, Audu R (2021). Validation of Commercial SARS-CoV-2 Immunoassays in a Nigerian Population. Microbiol Spectr..

[12] Ndagije HB, Manirakiza L, Kajungu D, Galiwango E, Kusemererwa D, Olsson S (2019). The effect of community dialogues and sensitization on patient reporting of adverse events in rural Uganda: Uncontrolled before-after study. PLoS One.

[13] Rath S, Nair N, Tripathy PK, Barnett S, Rath S, Mahapatra R, Gope R, Aparna B, Sinha R, Costello A, Audrey PA (2010). Explaining the impact of a women’s group led community mobilisation intervention on maternal and newborn health outcomes: the Ekjut trial process evaluation. BMC Int Health Hum Rights.

[14] Osrin D, Azad K, Fernandez A, Manandhar DS, Mwansambo CW, Tripathy P, Costello A. Ethical challenges in cluster randomized controlled trials: experiences from public health interventions in Africa and Asia. Bulletin of the World Health Organization; T Policy and Practice, 2009. 10.2471/BLT.08.051060. 10.2471/BLT.08.051060 PMC2755306 19876544

[15] Manandhar DS, Osrin D, Shrestha BP, Mesko N, Morrison J, Tumbahangphe KM, Tamang S, Thapa S, Shrestha D, Thapa B, Shrestha JR, Wade A, Borghi J, Standing H, Manandhar M, Costello AM, Members of the MIRA Makwanpur trial team (2004). Effect of a participatory intervention with women's groups on birth outcomes in Nepal: cluster-randomised controlled trial. Lancet (London, England)..

[16] Edgren KK, Parker EA, Israel BA, Lewis TC, Salinas MA, Robins TG, Hill YR. Community involvement in the conduct of a health education intervention and research project: Community Action Against Asthma. Health Promot Pract. 2005;6(3):263–9. 10.1177/1524839903260696. 10.1177/1524839903260696 16020621

[17] World Medical Association (2001). Declaration of Helsinki Ethical principles for medical research involving human subjects. Bull World Health Organ.

[18] USAID. Lessons learned in implementing the religious leaders RH/FP programme: A case study of Dadaab refugee camp. The Communication Initiative Network website. 2008. Available online at: https://www.comminit.com/content/lessons-learned-programming-and-implementing-religious-leaders-rhfpprogramme-case-dadaa. Accessed 28 Aug 2022.

[19] Dwarakanathan V, Kumar A, Nongkynrih B, Kant S, Gupta SK (2018). Challenges in the conduct of community-based research. Speaking for Myself. Natl Med J India.

[20] Gwadabe NM, Balogun AD. The challenges and strategies of accessing hard to reach locations during fieldwork data collection: the case of northeast Nigeria2021. 10.4337/9781800376328.00015.

[21] Adamu A, Rasheed ZH (2016). Effects of insecurity on the internally displaced persons (IDPs) in Northern Nigeria: prognosis and diagnosis. Glob J Hum Soc Sci.

