# Supplementary material for: Challenges in conducting population-based seroepidemiology survey of COVID-19 in Lagos State, Nigeria
Source: BMC Public Health. 2023 Dec 21;23:2559. doi: 10.1186/s12889-023-17125-1 (PMC10740330; doi:10.1186/s12889-023-17125-1)
Supplement: Supplementary file 1 — Additional file 1. [file 12889_2023_17125_MOESM1_ESM.zip › IRB APPROVAL.pdf]

NIGERIAN INSTITUTE OF MEDICAL RESEARCH
INSTITUTIONAL REVIEW BOARD

6, Edmund Crescent, off Muritala Mohammed Way, PMB 2013, Yaba, Lagos. Nigeria.
Tel: +234 0909 016 6992, +234 0909 213 3886 **Email:** nimr_irb@yahoo.com **Website:** www.nimr.gov.ng
Secretariate: Ground Floor, Laboratory Complex

1st Dec. 2020

PROJECT TITLE: POPULATION-BASED SEROEPIDEMIOLOGICAL AND HOUSEHOLD CONTACT STUDY OF COVID-19 VIRUS INFECTION IN NIGERIA.

NIMR - INSTITUTIONAL REVIEW BOA
Protocol No: IRB-20-104
Effective: 1st DEC 2020
Expiration: 30th NOV 2021
Sign:

PROJECT №: IRB/20/104

APPROVAL LETTER

The above named proposal has been adequately reviewed; the protocol and safety guidelines satisfy the conditions of NIMR-IRB policies regarding experiments that use human subjects.

Therefore the study under its reviewed state is hereby approved by Institutional Review Board, NIMR.

Prof. O. C. Ezechi 01/12/2020
Name of IRB Chairman — *Signature of IRB Chairman & Date*

Dr. O. A. NWOGBE 01/12/2020
Name of IRB Secretary — *Signature of IRB Secretary & Date*

INSTITUTIONAL REVIEW BOARD NIMR Sign & Date: APPROVAL

This approval is given with the investigator's Declaration as stated below;
By signing below I agree/certify that:

1. I have reviewed this protocol submission in its entirety and that I am fully cognizant of, and in agreement with, all submitted statements.
2. I will conduct this research study in strict accordance with all submitted statements except where a change may be necessary to eliminate an apparent immediate hazard to a given research subject.
   - I will notify the IRB promptly of any change in the research procedures necessitated in the interest of the safety of a given research subject.
   - I will request and obtain IRB approval of any proposed modification to the research protocol or informed consent document(s) prior to implementing such modifications.

Dr. Oliver C. Ezechi - Chairman **Dr. Agatha N. David** - Vice Chairman **Mrs. Olayemi A. Nwogbe** - Secretary **Dr. Tajudeen Bamidele** - Assistant Secretary

3. I will ensure that all co-investigators and other personnel assisting in the conduct of this research study have been provided a copy of the entire current version of the research protocol and are fully informed of the current (a) study procedures (including procedure modifications); (b) informed consent requirements and process; (c) potential risks associated with the study participation and the steps to be taken to prevent or minimize these potential risks; (d) adverse event reporting requirements; (e) data and record-keeping; and (f) the current IRB approval status of the research study.

4. I will respond promptly to all requests for information or materials solicited by the IRB or IRB Office.

5. I will submit the research study in a timely manner for IRB renewal approval.

6. I will not enrol any individual into this research study until such time that I obtain his/her written informed consent, or, if applicable, the written informed consent of his /her authorized representative (i.e., unless the IRB has granted a waiver of the requirement to obtain written informed consent).

7. I will employ and oversee an informed consent process that ensures that potential research subjects understand fully the purpose of the research study, the nature of the research procedures they are being asked to undergo, the potential risks of these research procedures, and their rights as a research study volunteer.

8. I will ensure that research subjects are kept fully informed of any new information that may affect their willingness to continue to participate in the research study.

9. I will maintain adequate, current, and accurate records of research data, outcomes, and adverse events to permit an on-going assessment of the risks/benefit ratio of research study participation.

10. I am cognizant of, and will comply with, current federal regulations and IRB requirements governing human subject research including adverse event reporting requirements.

11. I will make a reasonable effort to ensure that subjects who have suffered an adverse event associated with research participation receive adequate care to correct or alleviate the consequences of the adverse event to the extent possible.

12. I will ensure that the conduct of this research study adheres to Good Clinical Practice guidelines.

**Prof R. A Audu**	.... 01/02/2021
Principal Investigator's Name	Principal Investigator's Signature and Date
