# Supplementary material for: Challenges in conducting population-based seroepidemiology survey of COVID-19 in Lagos State, Nigeria
Source: BMC Public Health. 2023 Dec 21;23:2559. doi: 10.1186/s12889-023-17125-1 (PMC10740330; doi:10.1186/s12889-023-17125-1)
Supplement: Supplementary file 1 — Additional file 1. [file 12889_2023_17125_MOESM1_ESM.zip › NHREC Approval for COVID-19 Seroepidemilogical Study in Nigeria.pdf]

National Health Research Ethics Committee of Nigeria (NHREC)

Promoting Highest Ethical and Scientific Standards for Health Research in Nigeria

Federal Ministry of Health

NHREC Protocol Number NHREC/01/01/2007-02/05/2020
NHREC Approval Number NHREC/01/01/2007-14/05/2020
Date: 14 May, 2020

Re: Population –based Seroepidemiological and household contact study of COVID-19 Virus infection in Nigeria

Health Committee assigned number: NHREC/01/01/2007
Name of Principal Investigator: Prof. Rosemary Audu
Address of Principal Investigator: Director of Research
Head, Microbiology Department
Nigerian Institute of Medical Research
Email: rosemaryaudu@gmail.com
Tel: +2348035017790; +2348050422304
Date of receipt of valid application: 02/05/2020
Date when final determination of research was made: 14-05-2020

Notice of Expedited Committee Review and Approval

This is to inform you that the research described in the submitted protocol, the consent forms, advertisements and other participant information materials have been reviewed and *given expedited committee approval by the National Health Research Ethics Committee.*

This approval dates from 14/05/2020 to 13/05/2021. If there is delay in starting the research, please inform the HREC so that the dates of approval can be adjusted accordingly. Note that no participant accrual or activity related to this research may be conducted outside of these dates. *All informed consent forms used in this study must carry the HREC assigned number and duration of HREC approval of the study.* In multiyear research, endeavour to submit your annual report to the HREC early in order to obtain renewal of your approval and avoid disruption of your research.

The National Code for Health Research Ethics requires you to comply with all institutional guidelines, rules and regulations and with the tenets of the Code including ensuring that all adverse events are reported promptly to the HREC. No changes are permitted in the research without prior approval by the HREC except in circumstances outlined in the Code.

The HREC reserves the right to conduct compliance visit to your research site without previous notification.

Signed

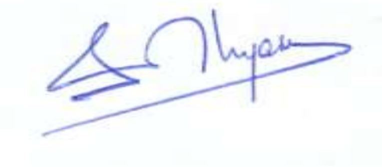

Professor Zubairu Iliyasu MBBS (UniMaid), MPH (Glasg.), PhD (Shef.), FWACP, FMCPH
Chairman, National Health Research Ethics Committee of Nigeria (NHREC)

Department of Health Planning, Research & Statistics
Federal Ministry of Health
11th Floor, Federal Secretariat Complex Phase III
Ahmadu Bello Way, Abuja

Tel: +234-09-523-8367
E-mail: chairman@nhrec.net, secretary@nhrec.net, deskofficer@nhrec.net,
URL: http://www.nhrec.net,
